# Prevalence and risk factors of mental distress in China during the outbreak of COVID‐19: A national cross‐sectional survey

**DOI:** 10.1002/brb3.1818

**Published:** 2020-09-01

**Authors:** Mindan Wu, Huanqin Han, Tingkui Lin, Min Chen, Jun Wu, Xufei Du, Guomei Su, Dong Wu, Fagui Chen, Qichuan Zhang, Hailin Zhou, Dan Huang, Bin Wu, Jiayuan Wu, Tianwen Lai

**Affiliations:** ^1^ Department of Respiratory and Critical Care Medicine Affiliated Hospital of Guangdong Medical University Zhanjiang China; ^2^ Department of Respiration Shantou Central Hospital Affiliated Shantou Hospital of Sun Yat‐sen University Shantou China; ^3^ Department of Infectious Diseases Center Affiliated Hospital of Guangdong Medical University Zhanjiang China; ^4^ Department of Hospital Office Affiliated Hospital of Guangdong Medical University Zhanjiang China; ^5^ Key Laboratory of Respiratory Disease of Zhejiang Province Department of Respiratory and Critical Care Medicine Second Affiliated Hospital of Zhejiang University School of Medicine Hangzhou China; ^6^ Department of Clinical Research Affiliated Hospital of Guangdong Medical University Zhanjiang China

**Keywords:** anxiety, COVID‐19, depression, psychological distress, structural equation modeling

## Abstract

**Objective:**

As a result of the pandemic of COVID‐19, the public have been experiencing psychological distress. However, the prevalence of psychological distress during the COVID‐19 pandemic remains unknown. Our objective was to evaluate the prevalence of psychological distress during COVID‐19 outbreak and their risk factors, especially their internal paths and causality.

**Methods:**

A nationwide cross‐sectional survey of the prevalence of mental disorders was conducted. We used Hospital Anxiety and Depression Scale (HADS) to estimate the prevalence of anxiety and depression. The internal paths and the causality of the psychological health were analyzed using a structural equation modeling (*SEM*) approach.

**Results:**

A total of 24,789 respondents completed the survey. We found that the overall prevalence of anxiety, depression, combination of anxiety, and depression were 51.6% (95% CI: 51.0–52.2), 47.5% (95% CI: 46.9–48.1), and 24.5% (95% CI: 24.0–25.0), respectively. The risk of psychological disorders in men was higher than that in women. The status of psychological health was different across different age groups, education levels, occupations, and income levels. The *SEM* analysis revealed that inadequate material supplies, low income, low education, lack of knowledge or confidence of the epidemic, and lack of exercise are major risk factors for psychological distress.

**Conclusions:**

The evidence from this survey poses serious challenges related to the high prevalence of psychological distress, but also offers strategies to deal with the mental health problems caused by the COVID‐19 pandemic.

## Graphical abstract

In national cross‐sectional survey with largest participants in china, we pose serious challenges related to the high prevalence of psychological distress, find out major risk factors for them, and then offer evidence‐based strategies to deal with the mental health problems caused by the COVID‐19 pandemic. Our research also provides evidences for other countries suffering from the COVID‐19 to take public health interventions to relieve the public psychological pressure.

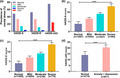

## 1 INTRODUCTION

In December 2019, novel coronavirus disease 2019 (COVID‐19) occurred in Wuhan, China and rapidly spread throughout China and around the world (Guan et al., 2020; Huang et al., 2020; Hui et al., 2020; Lu et al., 2020). Until May 2, 2020, 84,388 confirmed cases of COVID‐19 infection have been reported in China (4,643 deaths) and 3,182,796 in 215 countries/areas/territories outside of China (225,328 deaths) with overall mortality rates of 7.03% (WHO). The number of deaths worldwide from the new coronavirus overtook the fatalities caused by the severe acute respiratory syndrome (SARS) in 2003, which was the most serious infectious disease outbreak in China prior to the COVID‐19 (Guan et al., 2020). The increasing number of confirmed and death cases of the COVID‐19, and more and more countries affected by the epidemic, have raised public concerns about infection. A high‐intensity stressful life event is an important stressor that triggers individual psychological disorder. COVID‐19 outbreak has caused public panic and mental health stress in China. Rumors and misinformation, which often caused by erroneous information and misunderstandings of the epidemic, have exacerbated the unpredictable future of COVID‐19 outbreak and resulted in public panic (Duan & Zhu, 2020). In order to prevent and control the spread of COVID‐19, China government has taken many measures including forced quarantines and city‐wide lockdowns (Rosling & Rosling, 2003). These measures may cause public anxiety and depression when trying to control the COVID‐19 outbreak. Moreover, some factors such as inadequate material supplies, businesses, and travel shut down, may also cause psychological problems.

Previous studies declared that psychiatric morbidity was significantly increased during outbreaks of infection (Verghese, 2004). During the SARS epidemic, the prevalence of SARS‐related psychiatric was 22.9% and the presence of psychiatric morbidity was associated with younger age, less substance use, and increased self‐blame (Sim, Chan, Chong, Chua, & Soon, 2010). About 10% to 30% of the general public were very or fairly worried about the possibility of contracting the virus during one influenza outbreak (Rubin, Potts, & Michie, 2010).

As a result of the rapidly increasing numbers of confirmed cases and deaths, the National Health Commission of China has issued several guideline documents for emergency psychological crisis intervention for the COVID‐19 epidemic (Nickell et al., 2004). However, the effect of COVID‐19 outbreak on mental health remains unknown. To date, there are no nationwide studies with a large sample on psychological status during COVID‐19 outbreak in China. In particular, understanding the determinants of psychological disorders and their interactions may provide the basis for formulating public health interventions to deal with psychological distress during COVID‐19 outbreak. Therefore, a nationwide cross‐sectional study was conducted in the present study to describe the prevalence of psychological distress in the general population during COVID‐19 outbreak in China, and identified risk factors associated with psychological distress and their internal path and causality.

## 2 MATERIALS AND METHODS

### 2.1 Design and participants

This cross‐sectional nationwide study was conducted through online survey based on the Wenjuanxing platform (https://www.wjx.cn) during Feb 13, 2020 to Feb 29, 2020. The study was approved by the Research Ethics Committee of the Affiliated Hospital of Guangdong Medical University (PJ2020‐010).

### 2.2 Measurements

We used a validated Chinese version of Hospital Anxiety and Depression Scale (HADS) to estimate the prevalence of mental distress (Table S1). HADS includes two subscales: anxiety (HADS‐A) and depression (HADS‐D) and combination of anxiety and depression (HADS‐cAD). The total scores of each subscale range from 0 to 21 (a higher total score indicates a more severe symptom). The total score was divided into normal (< 8), mild (8–10), moderate (11–14), and severe (15–21) psychological disorder. (Mykletun, Stordal, & Dahl, 2001) All participants also reported their general characteristics and the effects of the COVID‐19 outbreak on their daily life. The descriptive statistics of the variables in the present survey are shown in Table S1.

### 2.3 Structural Equation Model Approach (*SEM*)

A structural equation model (*SEM*), which analyzes the relationship between variables based on their covariance matrix, was adopted to assess the causal assumption of the effects of various potential variables on psychological health during the COVID‐19 outbreak. An *SEM* includes a measurement model, which is applied to describe the linear relationships between observed variables and latent variables, and a structural model, while is used to represent the causal relationship between the latent variables (MacCallum & Austin, 2000). The factors affecting psychological health form a complex system. An *SEM* is useful for determining the hierarchy, path, and causal relationship of this system through path analysis, multiple linear regression analysis, and confirmatory factor analysis. In this study, latent variables and their measurement variables were set as follow: (a) psychological health, including anxiety and depression; (b) material supplies, including daily necessity supply (food, personal hygiene products, household items, kitchen and bath products, etc.), protective products supply (breathing mask, ethyl alcohol, protective clothing, etc.), and medical resource supply (medical treatment, medication, and health equipment, etc.). The other potential factors on psychological health included sex, age, occupation, income per month (income), highest level of education (education), knowledge about the COVID‐19 outbreak (knowledge), confidence in overcoming the COVID‐19 epidemic (confidence), and exercise during the COVID‐19 outbreak (exercise). Because all variables in this study conformed to normal distributions, a maximum likelihood method was used to estimate covariance parameters of initial model (Li & Zhou, 2020). The metrics used for goodness‐of‐fit were goodness‐of‐fit index (GFI), adjusted goodness‐of‐fit index (AGFI), normed fit index (NFI), comparative fit index (CFI), incremental fit index (IFI), root mean square residual (RMR), and root mean square error of approximation (RMSEA). A model was considered to have a good fit when the GFI, AGFI, NFI, CFI, and IFI were >0.90, RMR < 0.05, and RMSEA was <0.08.

### 2.4 Statistical analysis

All data analyses were conducted using SPSS (version 24.0) and AMOS (version 24.0) software (IBM Corporation). Because the status of the COVID‐19 epidemic varied in different regions, we divided the provinces, cities, or autonomous regions into five groups, namely high, high‐middle, middle, low‐middle, and low‐risk regions, according to the cumulative number of confirmed cases updated to Feb 29, 2020 to further investigate the effects of different epidemic situations on public psychological health. The high‐risk region included Hubei province where the epidemic was most severe, and the confirmed cases were more than 66,000. The provinces, cities, or autonomous regions had the cumulative number of confirmed cases more than 1,000 were classified into the high‐middle‐risk regions. The provinces, cities, or autonomous regions with the cumulative number of confirmed cases over 500 but <1,000 were divided into the middle‐risk regions. The low‐middle‐risk regions included the provinces, cities, or autonomous regions with confirmed cases >100 but <500. The low‐risk region included those provinces, cities, or autonomous regions with the cumulative number of confirmed cases less than 100. Descriptive analysis was performed to describe the included variables. We used logistic regression analysis to calculate the univariate associations between sociodemographic characteristics and the psychological health. An odds ratio (OR) of >1 with 95% confidence interval (CI) exceeding 1 indicated an increased risk of psychological disorders in this subgroup as compared to the reference group. A Pearson's correlation coefficient was used to evaluate the relationship between different parameters. A *p* value below .05 was regarded as statistical significance.

## 3 RESULTS

### 3.1 General characteristic of survey participants

Twenty four thousand nine hundred and twenty nine participants took part in our survey. After removing the participants without completed questionnaires (*n* = 140), 24,789 participants from 29 provinces and autonomous regions were involved. The characteristics of the respondents are summarized in Table 1. 13,304 (53.7%) respondents were male and 11,485 (46.3%) were female. Moreover, 5,298 respondents (21.4%) aged under aged 20 years, 7,993 (22.2%) aged 20–39 years, 5,487 (22.1%) aged 40–49, and 6,011 (24.3%) aged 60 years or older. Professional and technical staff (7,402, 29.9%) accounted for the highest proportion of the respondents, followed by students (5,955, 24.0%), self‐employed (5,281, 21.3%), and civil servant (5,402, 21.8%). More than half of the participants reported under bachelor or monthly income <2,000 China Yuan.

**Table 1:** Characteristics and psychological health of the study participants

Category	Total (*n*)	Anxiety			Depression			Anxiety and depression		
		*n*	Proportion (%, 95% CI)	OR (95% CI)	*n*	Proportion (%, 95% CI)	OR (95% CI)	*n*	Proportion (%, 95% CI)	OR (95% CI)
Overall	24,789	12,782	51.6 (51.0~52.2)		11,787	47.5 (46.9~48.1)		6,071	24.5 (24.0~25.0)	
Gender										
Male	13,304	7,052	53.0 (52.2~53.8)	1.13 (1.08~1.19)	6,292	47.3 (46.5~48.1)	0.98 (0.93~1.03)	3,354	25.2 (24.5~25.9)	1.09 (1.03~1.15)
Female	11,485	5,730	49.9 (49.0~50.8)	Reference	5,495	47.8 (46.9~48.7)	Reference	2,717	23.7 (22.9~24.5)	Reference
Age (years)										
<20	5,298	1,874	35.8 (34.5~37.1)	0.96 (0.89~1.04)	2,266	42.8 (41.5~44.1)	0.85 (0.79~0.91)	902	17.1 (16.1~18.1)	0.51 (0.42~0.60)
20–39	7,993	1,325	16.6 (15.8~17.4)	0.50 (0.45~0.52)	1,483	18.6 (17.7~19.5)	0.44 (0.32~0.55)	660	8.3 (7.7~8.9)	0.36 (0.31~0.42)
40–59	5,487	2,124	38.7 (37.4~40.0)	0.94 (0.87~1.01)	1,222	22.3 (21.2~23.4)	0.52 (0.46~0.59)	724	13.2 (12.3~14.1)	0.49 (0.42~0.55)
≥60	6,011	3,477	57.8 (56.6~59.0)	Reference	2,838	47.2 (45.9~48.5)	Reference	1,612	26.8 (23.7~27.9)	Reference
Education										
Under bachelor	14,069	7,847	55.8 (55.0~56.6)	0.92 (0.80~1.07)	7,138	50.7 (49.9~51.5)	3.64 (3.07~4.31)	3,993	28.4 (27.7~29.1)	2.74 (2.22~3.38)
Bachelor	6,805	2,800	41.1 (39.9~42.3)	0.52 (0.44~0.59)	3,711	54.5 (53.5~55.7)	4.24 (3.57~5.03)	1,581	23.2 (22.2~24.2)	2.10 (1.69~2.60)
Master	3,099	1,664	53.7 (51.9~55.5)	0.85 (0.73~0.99)	758	24.5 (23.0~26.0)	1.14 (0.95~1.38)	394	12.7 (11.5~13.9)	1.01 (0.80~1.27)
Doctor	816	471	57.7 (54.3~61.1)	Reference	180	22.1 (19.3~24.9)	Reference	103	12.6 (10.3~14.9)	Reference
Occupation										
Student	5,955	3,085	51.8 (50.5~53.1)	0.98 (0.94~1.02)	3,057	51.3 (50.0~52.6)	1.01 (0.97~10.5)	1,590	26.7 (25.6~27.8)	0.98 (0.93~1.03)
Professional and technical staff	7,402	4,230	57.1 (56.0~58.2)	1.03 (0.97~1.09)	2,769	37.4 (36.3~38.5)	0.61 (0.58~0.65)	1,592	21.5 (20.6~22.4)	0.84 (0.79~0.90)
Self‐employed	5,281	2,640	50.0 (48.6~51.2)	0.96 (0.91~1.01)	2,786	52.8 (51.1~54.5)	1.02 (0.96~1.08)	1,279	24.2 (23.2~25.2)	0.89 (0.81~0.97)
Civil servant	5,402	2,439	45.1 (43.7~46.5)	0.69 (0.60~0.78)	2,793	51.7 (49.8~53.6)	1.01 (0.97~1.05)	1,404	26.0 (24.7~27.3)	0.95 (0.88~1.03)
Others	749	398	53.1 (51.7~54.5)	Reference	382	51.0 (49.5~52.5)	Reference	206	27.5 (26.3~28.7)	Reference
Income per month (CNY)^a^										
<2,000	12,810	7,057	55.1 (54.2~56.0)	1.01 (0.97~1.05)	6,444	50.3 (49.4~51.2)	2.87 (2.22~3.50)	3,213	25.1 (24.3~25.9)	1.15 (1.03~1.28)
2,000–5.000	3,921	1,901	48.5 (46.9~50.1)	0.76 (0.68~0.83)	2,005	51.1 (49.5~52.7)	2.93 (2.27~3.58)	914	23.3 (22.0~24.6)	1.05 (0.98~1.13)
5,001–10,000	4,083	1,879	46.0 (44.5~47.5)	0.73 (0.66~0.82)	1,624	39.8 (38.3~41.3)	1.15 (1.05~1.24)	1,048	25.7 (24.4~27.0)	1.21 (1.08~1.34)
10,001–15,000	1,636	649	39.7 (37.3~42.1)	0.55 (0.47~0.63)	746	45.6 (43.2~48.0)	2.11 (1.63~2.62)	382	23.3 (21.3~25.3)	1.05 (0.94~1.18)
15,001–20,000	1,450	809	55.8 (53.2~58.4)	1.03 (0.98~1.07)	667	46.0 (43.4~48.6)	2.19 (1.71~2.70)	314	21.6 (19.5~23.7)	0.96 (0.88~1.04)
>20,000	889	487	54.8 (51.5~58.1)	Reference	301	33.9 (30.8~37.0)	Reference	200	22.5 (19.8~25.2)	Reference

^a^ CNY, China Yuan (1 CNY = 0.1413 USA dollar) (Update time: 2020‐03‐24).

### 3.2 Psychological status during the COVID‐19 epidemic

**Figure 1:**
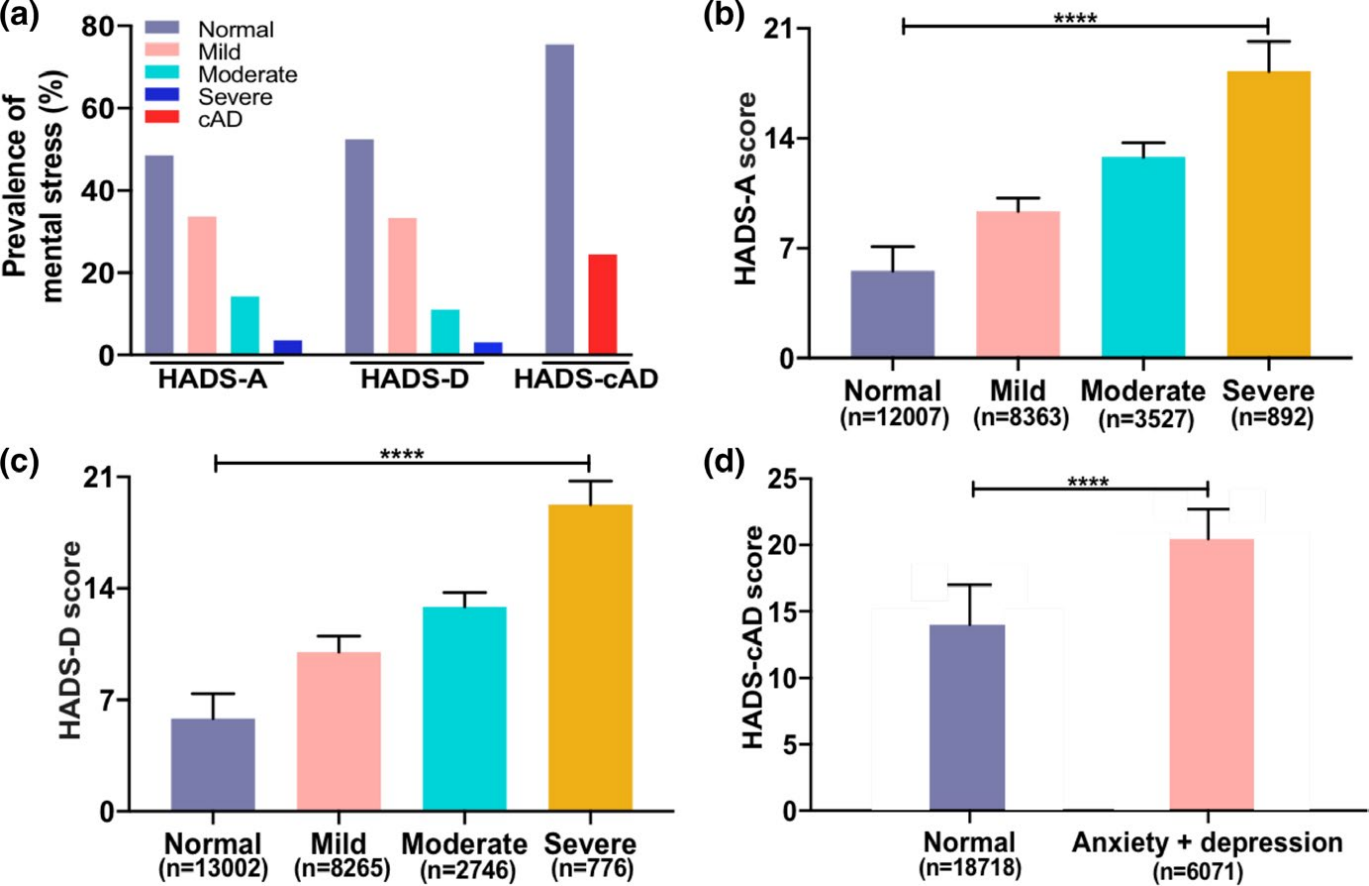
Prevalence of psychological disorders during COVID‐19 epidemic in China. (a) Percentage distributions of psychological disorders of different severity. (b) Hospital Anxiety and Depression Scale score among people with different severity of anxiety. (c) Hospital Anxiety and Depression Scale score among people with different severity of depression. (d) Hospital Anxiety and Depression Scale score among people with or without combined anxiety and depression. Note: Asterisks indicate a statistical significance of between‐group comparison according to the ANOVA variance analysis or *t* test (****p < .0001). HADS, Hospital Anxiety and Depression Scale; HADS‐A, HADS‐anxiety; HADS‐cAD, HADS‐comorbid anxiety and depression; HADS‐D, HADS‐depression

The nationwide prevalence of HADS‐A, HADS‐D, and HADS‐cAD during COVID‐19 outbreak was 51.6% (95% CI: 51.0–52.2), 47.5% (95% CI: 46.9–48.1), and 24.5% (95% CI: 24.0–25.0), respectively (Table 2). In terms of HADS‐A, 12,007 (48.4%) participants had no anxiety symptom with a mean score of 5.54 ± 1.56, 8,363 (33.7%) had mild symptom with a mean score of 9.33 ± 0.87, 3,527 (14.2%) had moderate symptom with a mean score of 12.79 ± 0.93, and 892 (3.6%) had severe symptom with a mean score of 18.26 ± 1.89. The number of participants with no, mild, moderate, and severe HADS‐D symptom were 13,002 (52.5%), 8,265 (33.3%), 2,746 (11.1%), and 776 (3.1%) with a mean score of 5.83 ± 1.55, 9.75 ± 0.79, 12.83 ± 0.92, and 19.25 ± 1.49, respectively. Moreover, 6,071 (24.5%) participants were rated as HADS‐cAD with a mean score of 19.45 ± 2.26 (Figure 1).

**Table 2:** Correlation matrix of model variables

Variable	Sex	Age	Education	Occupation	Income	Knowledge	Confidence	Exercise	Daily necessity	Protective supply	Medical resource	Anxiety	Depression
Sex	1.000												
Age	−0.059***	1.000											
Education	0.037***	0.035***	1.000										
Occupation	−0.022**	0.087***	0.143***	1.000									
Income	0.049***	0.006	0.631***	0.028***	1.000								
Knowledge	0.012	−0.065***	0.114***	−0.010	0.110***	1.000							
Confidence	−0.052***	0.080***	0.128***	0.077***	0.136***	0.133***	1.000						
Exercise	−0.005	0.021**	0.146***	−0.036***	0.144***	0.054***	0.073***	1.000					
Daily necessity	−0.033***	0.089***	0.219***	0.086***	0.244***	−0.040***	0.138***	−0.014*	1.000				
Protective supply	−0.032***	0.066***	0.058***	−0.006	0.055***	0.114***	0.164***	−0.076***	0.055***	1.000			
Medical resource	−0.055***	0.098***	0.145***	−0.138***	0.173***	−0.019***	−0.008	0.045***	0.221***	0.132***	1.000		
Anxiety	−0.031***	0.076***	0.142***	−0.088***	0.183***	0.035***	0.201***	0.096***	0.189***	0.228***	0.259***	1.000	
Depression	0.006	−0.041***	0.154***	0.051***	0.205***	0.038***	0.158***	0.040**	0.233***	0.161***	0.149***	0.211***	1.000

Note

Knowledge: knowledge of the COVID‐19; Confidence: confidence in fight against the COVID‐19; Exercise: exercise during the outbreak.

* *p* < .05

** *p* < .01

*** *p* < .001

As shown in Table 1, the risk of HADS‐A (OR = 1.13, 95% CI: 1.08–1.19) and HADS‐cAD (OR = 1.09, 95% CI: 1.03–1.15) was higher in men than those in women. The risk of psychological disorders was different among different age groups. As compared to people aged over 60 years, the younger people had a lower risk of HADS‐D and HADS‐cAD, and people aged 20–39 years had a lower risk of anxiety. People with different education levels showed different psychological status during the COVID‐19 epidemic, with the higher risk of HADS‐D and HADS‐cAD was observed in people with lower degrees as compared to people with doctor degree. However, people with lower education levels had a lower risk of HADS‐A as compared to those with doctor degree. Moreover, the psychological status during the COVID‐19 outbreak varied among people with different occupations or income levels.

According to the epidemic status of COVID‐19, the incidence of HADS‐A and HADS‐cAD was observed in the middle‐risk regions, followed by the low‐ and low‐middle‐risk regions (Table S2). However, the incidence of HADS‐D was found in the high‐risk regions, followed by high‐middle‐ and low‐risk regions.

**Figure 2:**
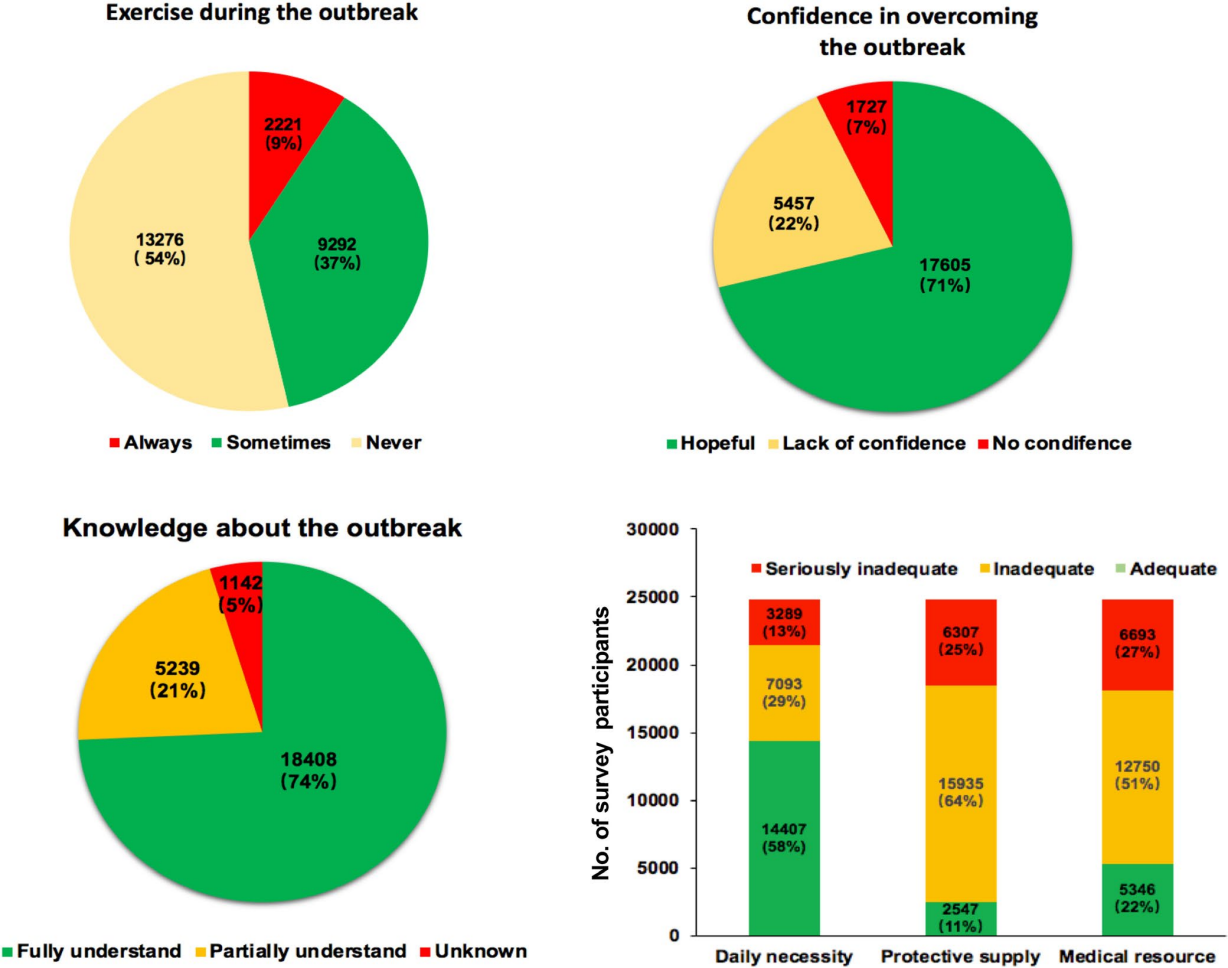
Effects of the COVID‐19 outbreak on public daily life. (a) exercise during the COVID‐19 outbreak; (b) confidence in overcoming the COVID‐19 outbreak; (c) knowledge about the COVID‐19 outbreak; (d) material support

We next assessed the impact of COVID‐19 outbreak on people's daily life in four aspects including exercise, confidence, knowledge, and material supplies. First, 13,276 (54%) participants reported never do exercise, 9,292 (37%) reported sometimes do exercise, and only 2,221 (9%) reported always do exercise. Second, 17,605 (71%) participants reported confidence in fight against the COVID‐19 epidemic. Third, 18,408 (74%) participants reported understood the knowledge of the COVID‐19. Finally, 14,407 (58%) participants reported adequate daily necessity, whereas adequate supplies of protective products and medical resources were only reported in 2,547 (11%) and 5,346 (22%) cases, respectively (Figure 2).

### 3.3 Correlation analysis between psychological disorders and potential risk factors

We next determined the risk factors associated with psychological metal problems. The correlation coefficient matrix of various variables was showed in Table 2. HADS‐A was positively associated with age, education, knowledge, income, confidence, exercise, and material supplies (daily necessity, protective supplies, and medical resources), but was negatively associated with gender and occupation. HADS‐D had a positive significant correlation with education, occupation, income, knowledge, confidence, exercise, and material supplies, whereas a negative significant correlation with age.

### 3.4 *SEM* analysis of the impact of the potential determinants on psychological health

We further assessed the results of correlation analyses using *SEM* analysis. Some variables including gender, age, and occupation were not included as covariates in *SEM* analysis because they showed relatively low correlations with all other variables (< 0.01). By combining the previously observed interactions between variables with a broad literature review, a priori hypothesized model was constructed based on the following hypotheses: (1) income, education, knowledge, confidence, exercise, and material supplies, have direct influence on psychological health; (2) income have indirect impacts on psychological health mediated by material supplies; (3) education, knowledge, and exercise have indirect effects on psychological health with confidence as the mediation (Figure S1).

An evaluation of the overall goodness‐of‐fit of the *SEM* models was conducted to determine its suitability for analyzing the effect of the COVID‐19 outbreak on psychological health (Table 3). All fitting indexes of the initial model were far from the measurement standards, indicating the data failed to support the theoretical model. According to the modification index, two pair of covariance parameters between education and exercise, and material supplies and confidence should be placed. Moreover, covariance parameters should be included in the model due to these correlations in line with theoretical considerations. After adjusted, most of the fitting indexes were within or close to the reasonable range, indicating that the final model's construction was reasonable and the fitness was good.

**Table 3:** Evaluation of the overall goodness‐of‐fit of the *SEM*

Parameters	Initial model	Final model	Measurement standard
GFI	0.818	0.922	>0.90
AGFI	0.797	0.913	>0.90
NFI	0.812	0.905	>0.90
CFI	0.777	0.893	>0.90
IFI	0.745	0.893	>0.90
RMR	0.086	0.040	<0.05
RMSEA	0.68	0.060	<0.08

Abbreviations: AGFI, adjusted goodness‐of‐fit index; CFI, comparative fit index; GFI, goodness‐of‐fit index; IFI, incremental fit index; NFI, normed fit index; RMR, root mean square residual; RMSEA, root mean square error of approximation.

**Figure 3:**
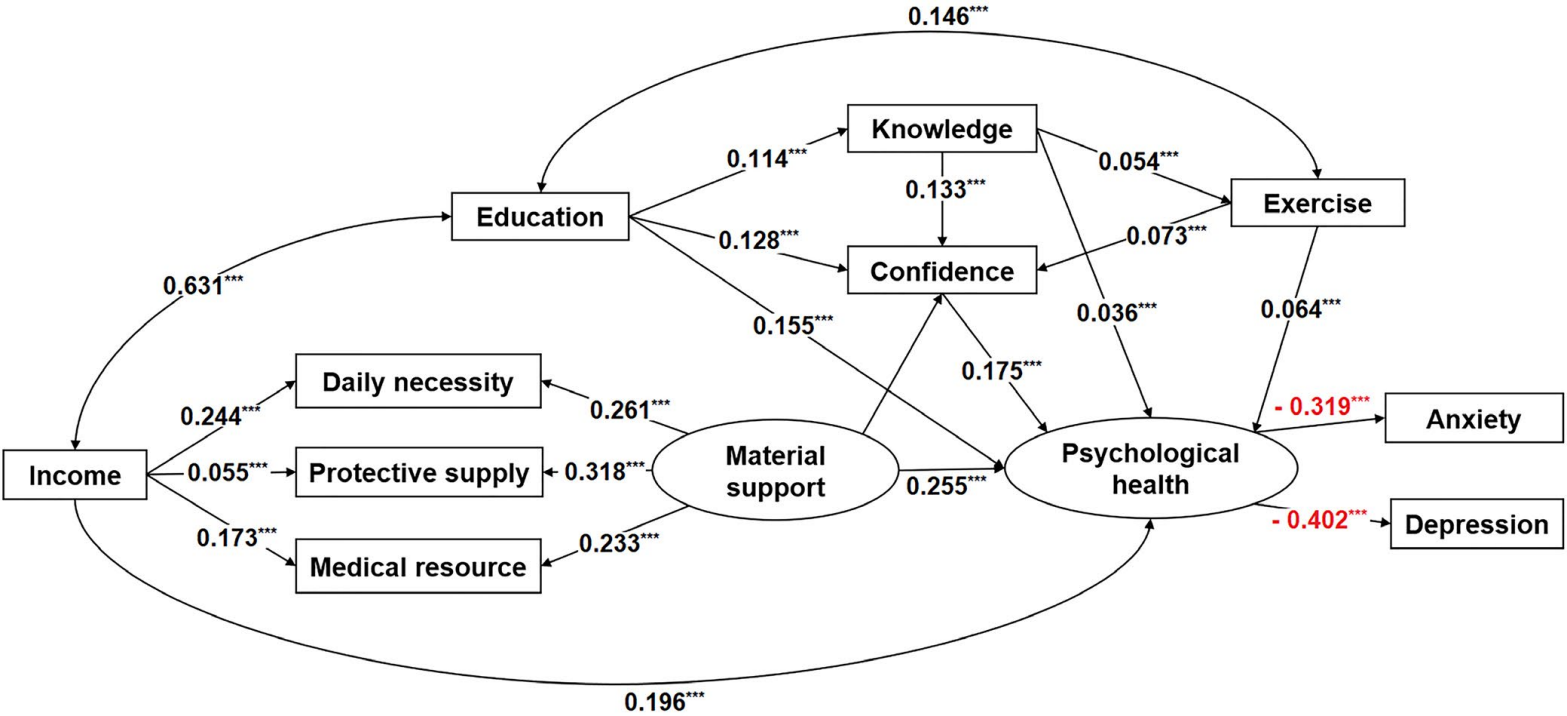
Results of the *SEM* path diagram. Ellipses represent latent variables and rectangles represent observed variables. Numbers represent the standardized path coefficients. *p < .1, **p < .05, ***p < .001. Exercise: exercise during the outbreak; Confidence: confidence in fight against the COVID‐19; Knowledge: knowledge of the COVID‐19

The result of the final *SEM* path diagram was showed in Figure 3. Income, education, knowledge, confidence, exercise, and material supplies had a significant direct positive effects on standardized coefficients of 0.196, 0.155, 0.036, 0.175, 0.064, and 0.255, respectively. These results indicated that for every one standard deviation increase in these parameters, psychological health increases by 0.196, 0.155, 0.036, 0.175, 0.064, and 0.255, respectively. Beside a direct influence, income also had an indirect impact on psychological health. That is, income significantly influences material supplies and then material supplies positively and significantly influences psychological health. In addition, material supplies, education, knowledge, and exercise also had indirect and significant positive impacts on psychological health with confidence as the mediation.

## 4 DISCUSSION

To our knowledge, this is the first large‐scale nationwide survey of mental disorders during the COVID‐19 epidemic. We found that the prevalence of HADS‐cAD was (24.5%, 95% CI: 24.0–25.0) and much higher prevalence of HADS‐A (51.6%, 95% CI: 51.0–52.2 vs. 7.6%, 95% CI: 6.3–8.8) and HADS‐D (47.5%, 95% CI: 46.9–48.1 vs. 6.9%, 95% CI: 6.6–7.2) than those reported in a latest national survey of general psychological status in 2015 (Huang et al., 2019). The impact of the COVID‐19 epidemic on psychological health is multifactorial, which can be conceptualized into a mediation framework. Psychological distress was significantly associated with material supplies, income, education level, knowledge of the epidemic, confidence in fighting against the epidemic, and exercise. Our findings pose serious challenges related to the high prevalence of psychological distress, but also provide valuable strategies for policy makers and physicians to identify and address the factors that affect psychological healths during the COVID‐19 epidemic.

The outbreak of the coronavirus is a huge public health concern across the world. COVID‐19 was first reported in Wuhan, Hubei Province and has subsequently spread to other regions of China (Guan et al., 2020; Huang et al., 2020; Koh et al., 2005). The increasing number of confirmed cases and deaths has caused public panic and mental health stress in China. According to previous surveys on the public psychological status after public emergencies in China, such as SARS, H1N1 avian influenza, and Wenchuan earthquake, more than half of people have suffered from psychological problems (Lau, Griffiths, Choi, & Tsui, 2010; Tsang, Scudds, & Chan, 2004; Wu, Xu, & He, 2014). In the present study, the nationwide prevalence of HADS‐A, HADS‐D, and HADS‐cAD during the outbreak of COVID‐19 in China was 51.6%, 47.5%, and 24.5%, respectively. The prevalence of psychological problems varies in different provinces and autonomous regions. Concerning the worst‐hit Hubei province, the incidences of HADS‐A and HADS‐cAD were lower than the national average. By contrast, the incidences of psychological disorders were much higher in some provinces where the epidemic was less severe as compared to the regions with relatively higher risk. It seemed to be contradictory with the common sense that the worse the outbreak, the worse the psychological health. However, it was not difficult to explain this phenomenon, because it is common knowledge in psychology that the best way to overcome fear is to experience it. The people in the severe epidemic area may have more opportunities and motivation to learn about the outbreak; thus, they can receive factual feedback. Moreover, the nationwide support for Hubei province, such as the implementation of “one province support one city in Hubei”, has greatly enhanced the confidence of the people in Hubei to conquer the epidemic, from which the people can timely adjust their own emotion and receive a positive psychological feedback.

Moreover, we used *SEM* model to explore the casual relationship between the potential risk factors and psychological disorders. We found that low income, low education, and inadequate material supplies were risk factors for people with psychological distress during COVID‐19 outbreak. The reasons for this may be as follows. The inequality of socioeconomic status, such as low income and low education, is associated with higher risk of mental health problems (Daly, Boyce, & Wood, 2015; Gero, Kondo, Kondo, Shirai, & Kawachi, 2017; Schlax et al., 2019). Low socioeconomic status has a detrimental effect on health outcomes, as well as ability to use health resources. In contrast, higher income allows access to better quality material resources and better, easier or faster access to health services, which have a direct effect on mental health (Daly et al., 2015; Gero et al., 2017). Higher education enables people to cultivate self‐confidence and perceive control of anxiety and depression. Moreover, those with higher education and adequate knowledge about the COVID‐19 tend to do more excise to improve their physical fitness, which can help to keep a healthy emotion directly or through strengthening confidence. After the outbreak of the epidemic, especially since the implementation of more stringent prevention and control measures by governments, logistics and supplies were affected (Park, Cho, & Moore, 2018). Inadequate material support, especially medical supplies and protective items, leads to nervousness or panic and negatively affects the emotional reaction. This finding is consistent with those from previous studies that the shortage of basic supplies was positively related to anxiety and anger (Blendon, Benson, DesRoches, Raleigh, & Taylor‐Clark, 2004; Wilken et al., 2017). These results are meaningful and have practical implications when conceptualized into a mediation framework. The proposed framework could be used by the policy makers to produce effective mitigation measures for the general public.

Given to the higher prevalence of psychological disorders of the general public in China during the COVID‐19 epidemic, the timely effective interventions are urgently needed to mitigate the psychological impact. Here, we propose several evidence‐based suggestions according to our statistical analysis results.

### 4.1 Provide timely psychological assistance service

First, the public should recognize that the occurrence of emotions or behaviors associated with anxiety and depression is a common and normal response to COVID‐19. Such negative psychological impact is not needful to suppress deliberately or deny completely. Previous study declared that moderate negative emotions help people to be alert to the epidemic (Brooks et al., 2020). Second, the government should provide the general public with timely psychological health assessment and assistance measures. It is necessary to give full play to the advantages of Internet medical services and further improve the "Internet + medical health" service functions, including but not limited to online health assessment, health guidance, health education, psychological counseling, etc.

### 4.2 Provide the public with timely accurate knowledge

If government fails to release information in a timely, accurate, and comprehensive manner, it will provide conditions for the spread of grapevine news and rumors, resulting in the inconsistency of public information sources, and the increase of public psychological pressure (Caleo et al., 2018; Neria & Sullivan, 2011; Rosling & Rosling, 2003). Therefore, government is supposed to not only provide enough accurate knowledge about COVID‐19 but also need to establish information monitoring regulations, such as promoting social media platforms including Twitter, Facebook, and Tencent to establish the self‐supervision system to limit the release and spread of misinformation.

### 4.3 Provide adequate supplies

Material supplies are the basic guarantee to ensure the people's quality of life and disease prevention (Blendon et al., 2004; Wilken et al., 2017). The government needs to develop a comprehensive plan to ensure material supplies, including daily necessity, protective supply, and medical resource, are not exhausted by coordinate the provision and redistribution of supplies. Moreover, donations from varieties of parties are encouraged.

### 4.4 Advocate healthy moderate exercises and reliable daily schedule

Healthy exercises are beneficial not just physically but also psychologically (Weinstein, Maayan, & Weinstein, 2015). Proper exercises for recreational purpose at home may be an effective means to alleviate stress and therefore mitigate psychological impact. Furthermore, in order to maintain a regular life rhythm and maintain a good living condition, a reliable daily schedule is advocated to ensure moderate exercises, adequate sleep, healthy diet, and some program for study and entertainment.

## 5 LIMITATIONS

Several potential limitations should be mentioned. First, our study was designed as a cross‐sectional survey. Longitudinal survey is warrant in the future. Second, given to ongoing COVID‐19 epidemics, the online questionnaire approach was suitable for quick assessment, but its results were self‐reported that might be subject to respondent bias. Third, although we tried our best to control for many covariates, some residual confounding caused by unmeasured factors might remained.

## 6 CONCLUSIONS

There is high prevalence of mental health problems during COVID‐19 outbreak. The influence of the COVID‐19 outbreak on psychological health is multifactorial, which can be conceptualized into a mediation framework. Our findings provide the basis for formulating public health interventions to deal with the mental distress caused by the COVID‐19 pandemic. Future longitudinal studies on this topic are warranted to provide a more comprehensive understanding of this issue.

## CONFLICT OF INTEREST

The authors declare no conflict of interest.

## AUTHOR CONTRIBUTIONS

All authors contributed to the collection and interpretation of data and approved the final report. TW.L and MD.W conceived and designed the idea for the article, supervised and checked the analyses, and wrote the final manuscript. MD.W, J.W, and B.W assisted the coprincipal investigators in the design, implementation, and data analysis for the study. HQ.H, TK.L, M.C, J.W, XF.D, GM.S, D.W, FG.C, QC.Z, and D.H contributed to data collection in the fieldwork. TW.L, MD.W, and JY.W cleaned and checked the data, wrote the code, did the analysis for the study, and wrote the initial draft. TW.L, MD.W, HQ.H, and HL.Z did the revision of our study.

### Peer Review

The peer review history for this article is available at https://publons.com/publon/10.1002/brb3.1818.

## Supporting information

FigS1

TableS1

TableS2

## Data Availability

The data that support the findings of this study are available from the corresponding author upon reasonable request.
