# Supplementary figures and images for: Prevalence and risk factors of mental distress in China during the outbreak of COVID‐19: A national cross‐sectional survey
Source: Brain Behav. 2020 Sep 1;10(11):e01818. doi: 10.1002/brb3.1818 (PMC7667324; doi:10.1002/brb3.1818)

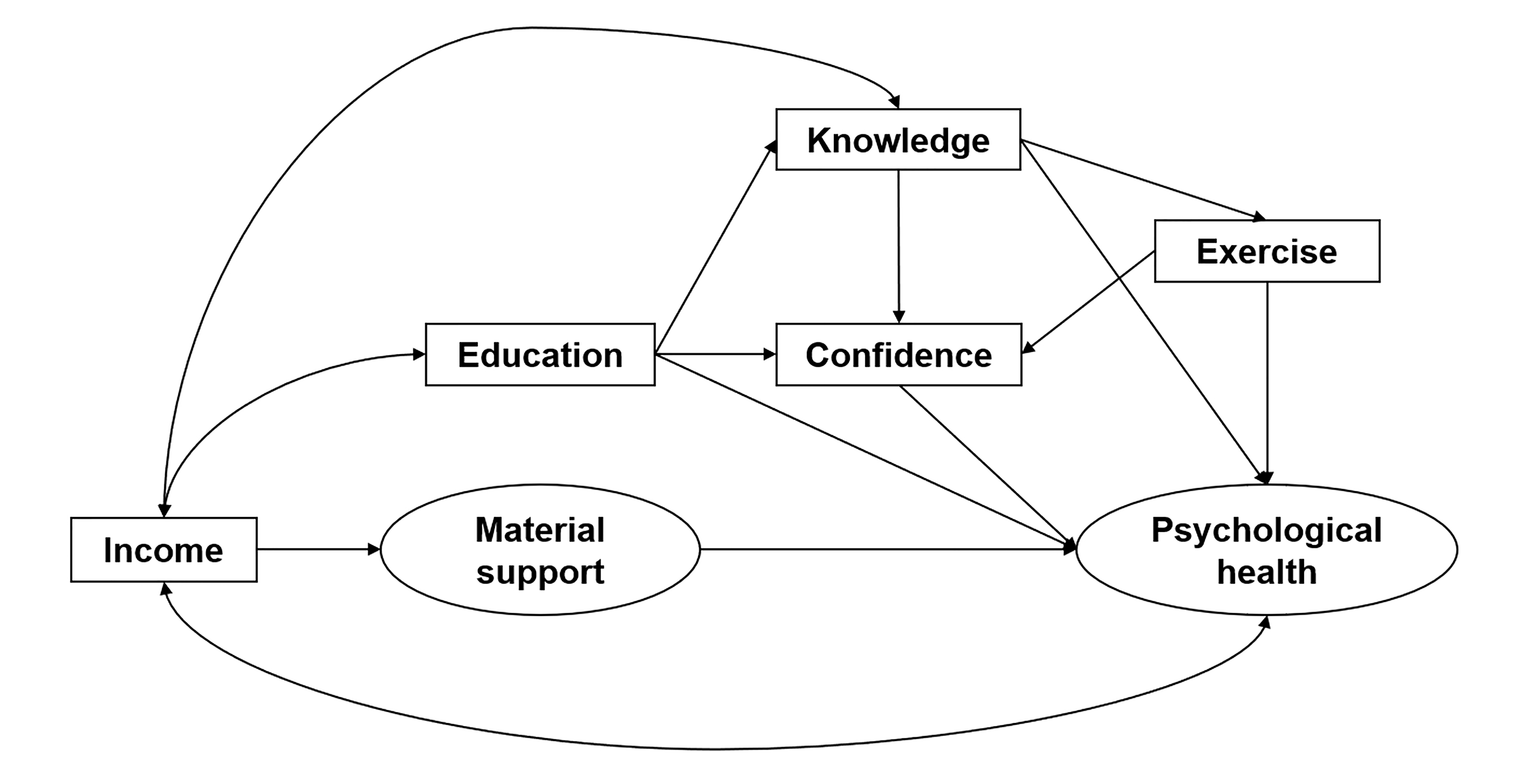

Supplement: Supplementary file 1 — FigS1 [file BRB3-10-e01818-s001.jpg]
