# Supplementary material for: Prevalence and risk factors of mental distress in China during the outbreak of COVID‐19: A national cross‐sectional survey
Source: Brain Behav. 2020 Sep 1;10(11):e01818. doi: 10.1002/brb3.1818 (PMC7667324; doi:10.1002/brb3.1818)
Supplement: Supplementary file 2 — TableS1 [file BRB3-10-e01818-s002.docx]

**Table S1. Descriptive statistics of variables**

| Variable | Description | Coding scheme | Mean | SD |
| --- | --- | --- | --- | --- |
| Sex | Sex | 1 = male  2 = female | 1.46 | 0.50 |
| Age | Age (years) | 1 < 20  2 = 20-39  3 = 40-59  4 > 60 | 3.46 | 1.91 |
| Occupation | Occupation | 1 = students  2 = professional and technical staff  3 = self-employed  4 = civil servant  5 = others | 3.31 | 1.17 |
| Education | Highest level of education | 1 = doctor  2 = master  3 = bachelor  4 = under bachelor | 1.62 | 0.83 |
| Income | Income per month (China Yuan) | 1 = > 20000  2 = 15001-20000  3 = 10001-15000  4 = 5001-10000  5 = 2000-5000  6 = < 2000 | 2.10 | 1.43 |
| Knowledge | Knowledge about the outbreak | 1 = fully understand  2 = partially understand  3 = unknown | 1.30 | 0.55 |
| Exercise | Exercise during the outbreak of NCP | 1 = always  2 = sometimes  3 = never | 2.45 | 0.65 |
| Daily necessity | Daily necessity supply (food, toiletries, household items, cooking supplies, etc.) | 1 = adequate  2 = inadequate  3 = seriously inadequate | 1.55 | 0.72 |
| Protective supply | Protective supply (breathing mask, ethyl alcohol, etc.) | 1 = adequate  2 = inadequate  3 = seriously inadequate | 2.15 | 0.72 |
| Medical resource | Medical resource supply (medical treatment, medication, etc.) | 1 = adequate  2 = inadequate  3 = seriously inadequate | 2.05 | 0.70 |
| Confidence | Confidence in overcoming the epidemic | 1 = hopeful  2 = lack of confidence  3 = no confidence | 1.36 | 0.61 |
| Anxiety | Anxiety | 1 = no  2 = yes | 1.52 | 0.50 |
| Depression | Depression | 1 = no  2 = yes | 1.48 | 0.50 |

SD, standard deviation.
