# Supplementary material for: Prevalence and risk factors of mental distress in China during the outbreak of COVID‐19: A national cross‐sectional survey
Source: Brain Behav. 2020 Sep 1;10(11):e01818. doi: 10.1002/brb3.1818 (PMC7667324; doi:10.1002/brb3.1818)
Supplement: Supplementary file 3 — TableS2 [file BRB3-10-e01818-s003.docx]

| **Table S2. The incidence rates of poor psychological status in regions with different epidemic status of COVID-19** | | | | | | | |
| --- | --- | --- | --- | --- | --- | --- | --- |
| **Region** | **No. of provinces**  **(no. of participants)** | **Anxiety** | | **Depression** | | **Anxiety and depression** | |
|  |  | ***n*** | **Incidence (%, 95% CI)** | ***n*** | **Incidence (%, 95% CI)** | ***n*** | **Incidence (%, 95% CI)** |
| China | 29 (24789) | 12782 | 51.6 (51.0~52.2) | 11787 | 47.5 (46.9~48.1) | 6071 | 24.5 (24.0~25.0) |
| High risk | 1 (287) | 112 | 39.0 (33.4~44.6) | 150 | 52.3 (46.5~58.1) | 56 | 19.5 (14.9~24.1) |
| High-middle risk | 4 (5622) | 2262 | 40.2 (38.2~42.2) | 2716 | 48.3 (46.2~50.4) | 1118 | 19.9 (18.3~21.5) |
| Middle risk | 6 (13686) | 7750 | 56.6 (55.5~57.5) | 6485 | 47.4 (46.3~48.5) | 3645 | 26.6 (25.6~27.6) |
| Low-middle risk | 13 (3717) | 1829 | 49.2 (46.9~51.5) | 1728 | 46.5 (44.2~48.8) | 864 | 23.2 (21.4~25.2) |
| Low risk | 5 (1447) | 829 | 56.1 (52.7~59.5) | 708 | 47.9 (44.5~51.3) | 388 | 26.3 (23.3~29.3) |
| Notes: high risk region included Hubei (cumulative number of confirmed cases updated to Feb 29, 2020: 66907); high-middle risk regions included Guangdong (1349), Henan (1272), Zhejiang (1205), and Hunan (1018); middle risk region included Anhui (990), Jiangxi (935), Shandong (756), Jiangsu (631), Chongqing (576), and Sichuan (538); low-middle risk region included Beijing (411), Shanghai (339), Heilongjiang (330), Hebei (318), Fujian (296), Guangxi (252), Shaanxi (245), Yunnan (174), Hainan (168), Guizhou (146), Tianjin (135), Shanxi (133), and Liaoning (122); low risk region included Jilin (93), Gansu (91), Xinjiang (76), Inner Mongolia (75), and Ningxia (69). | | | | | | | |
